# The importance of thinking about Guillain-Barré syndrome during the COVID-19 pandemic: a case with pure dysautonomic presentation

**DOI:** 10.1007/s13365-021-00997-7

**Published:** 2021-08-02

**Authors:** Erica Biassoni, Andrea Assini, Ilaria Gandoglia, Luana Benedetti, Silvia Boni, Emanuele Pontali, Marcello Feasi, Federica Gandolfo, Massimo Del Sette

**Affiliations:** 1 Department of Neuroscience, Rehabilitation, Ophthalmology, Genetics and Maternal and Child Health (DiNOGMI), University of Genoa, Largo Paolo Daneo 3, 16132 Genova, Italy; 2 IRCCS Ospedale Policlinico San Martino, Largo Rosanna Benzi 10, 16132 Genova, Italy; 3 Neurologic Unit, Galliera Hospital, Via Mura delle Cappuccine 14, 16128 Genova, Italy; 4 Department of Infectious Diseases, Galliera Hospital, Via Mura delle Cappuccine 14, 16128 Genova, Italy; 5 Geriatric Unit, Galliera Hospital, Via Mura delle Cappuccine 14, 16128 Genova, Italy

**Keywords:** Guillain-Barré syndrome, SARS-CoV-2, COVID-19 neurological complications, Dysautonomia

## Abstract

Guillain-Barré syndrome (GBS) is a peripheral nervous system disease caused by an immune-mediated inflammatory mechanism, usually triggered by a previous infectious process or vaccine; its typical presentation is a rapid and progressive bilateral limb hyposthenia, associated with sensory deficits and reduction or absence of osteotendinous reflexes. However, also autonomic nervous system can be involved with heart rate fluctuations, blood pressure instability, pupillary dysfunction, and urinary retention. Since the beginning of COVID-19 pandemic, GBS has been reported among neurological complications of SARS-CoV-2 infection, although etiopathological mechanisms still have to be clearly defined. We report the case of a 79-year-old man with multiple comorbidities, including diabetes, who was affected by SARS-CoV-2 interstitial pneumonia and developed dysautonomic symptoms after 10 days of hospitalization. A neurological evaluation was performed, and GBS was considered as a possible cause of the clinical manifestations. This hypothesis was confirmed by electrophysiological study and further supported, *ex-juvantibus*, by the satisfactory response to immunoglobulin treatment. In our opinion, this case of pure dysautonomic presentation of GBS in a SARS-CoV-2 positive patient is relevant because it suggests to consider GBS upon SARS-CoV-2 infection even if the symptoms have uncommon characteristics (e.g., pure vegetative manifestations) and if there are confounding factors which could lead to a misdiagnosis (e.g., old age, SARS-CoV-2 infection consequences and diabetes).

Guillain-Barré syndrome (GBS) is an immune-mediated inflammatory disease of the peripheral nervous system, usually preceded (4–6 weeks before) by viral infections or vaccinations (Leonhard et al. 2019). The most typical presentation is rapid and progressive bilateral symmetrical limb weakness associated with sensory deficits and reduced or absent reflexes, yet several clinical variants have been described. Weakness usually starts from lower limbs and progresses to upper limbs, cranial muscles and, in the 20% of cases, respiratory muscles. Involvement of the autonomic nervous system can also occur, causing heart rate fluctuations, blood pressure instability, pupillary dysfunction, and urinary retention (Leonhard et al. 2019). A variant of GBS, presenting as acute autonomic neuropathy, has also been reported (Koike et al. 2013).

Recently, GBS has been reported as a possible neurological complication of SARS-CoV-2 infection (Assini et al. 2020; Sriwastava et al. 2021), even if its mechanisms remain to be clarified (Ahmad and Rathore, 2020; Azizi and Azizi, 2020).

We report the case of a 79-year-old man, with diabetes and hypertension, who was admitted to our hospital because of thoracic pain, dyspnea, and fever. After chest computed tomography (CT) scan and nasopharyngeal swab, SARS-CoV-2 interstitial pneumonia was diagnosed. The patient was treated with oxygen, intravenous antiviral (remdesivir 200 mg on the first day, followed for the next 4 days by 100 mg), antibiotics (azithromycin and ceftriaxone), steroids, low molecular weight heparin, and insulin. After 10 days of hospitalization, respiratory symptoms progressively improved without require of intubation or vasopressor therapy (Metlay et al. 2019). Dysautonomic symptoms instead appeared, with acute urinary retention and several episodes of dizziness and reduction of level consciousness during orthostatism. Trans-thoracic echocardiography excluded valvular heart disease or other cardiac causes of syncope.

During lipothymic episodes, vital signs and glycemia were normal, although values of systolic blood pressure (BP) under 80 mmHg were recorded in orthostatism. Tilt test resulted positive: BP in supine position was 110/70 mmHg; in sitting position, after 1 min, BP decreased to 80/60 mmHg, and after 3 min, systolic BP lowered to 70 mmHg and patient began to blame dizziness. Therapy with midodrine, fludrocortisone, and elastic stockings was started.

Neurological examination was normal, except for absence of osteotendinous reflexes and mild confusional state.

Cerebral CT scan was normal, while neurophysiological examination, performed 6–7 days after symptoms onset, showed a reduction of conduction speed, temporal dispersion of potentials, late response, and F wave alterations, meaning both myelin and axonal damage (Table 1). We used the electrophysiological normative values of our neurophysiopathology laboratory, based on Kimura’s international references (Kimura, 2013). Cerebro-spinal fluid (CSF) analysis showed mild increase in proteins (45.30 mg/dl). Cell count was normal, and oligoclonal bands both in CSF and serum were negative. Search for SARS-CoV-2 virus and antibodies in CSF was negative, while serology for SARS-CoV-2 showed the presence of IgG but not IgM. Search for anti-ganglioside antibodies was negative (both IgG and IgM).

**Table 1:** Electrophysiological data

Examined nerves	Motor/sensory distal latency	Distal amplitude	Conduction velocity	F wave	Damage pattern
Left deep peroneal nerve	7.1 m (normal values less than 4.5 ms)	0.9 mV (normal values greater than 2.5 mV)	34.6 m/s (normal values greater than 40 m/s)	/	Mixed (distal and proximal demyelination and axonal motor damage)
Right posterior tibial nerve	4.2 ms (normal values less than 5.5 ms)	4.2 mV (normal values greater than 5 mV)	33.6 m/s (normal values greater than 40 m/s)	58 ms; poorly persistent (normal value: minimal latency of less than 56 ms)	Proximal demyelination and altered F wave
Right sural nerve	2.5 ms	1.5 mV (normal values greater than 5 µV)	42.7 m/s (normal values greater than 40 m/s)	/	Axonal damage
Left superficial peroneal nerve	Not evoked	Not evoked (normal values greater than 5 µV)	Not evoked (normal values greater than 40 m/s)	/	Axonal damage
Right median nerve	8.3 ms (normal values less than 4.2 ms)	1.1 mV (normal values greater than 5 mV)	Conduction block between elbow and wrist (normal values between elbow and wrist greater than 50 m/s)	Not evoked (normal value: minimal latency of less than 32 ms)	Mixed (proximal and distal demyelination with conduction block and axonal motor damage)

Given the hypothesis of GBS, 9 days after dysautonomic symptom onset, therapy with intravenous immunoglobulins was started, with partial improvement of dysautonomic symptoms. The Modified Erasmus GBS Outcome Score (mEGOS) was 2 (Waalgard et al. 2011). After about 28 days of hospitalization, SARS-CoV-2 swab turned negative and the patient was transferred to a rehabilitation center.

During rehabilitation, orthostatic hypotension improved and about 4 weeks after hospital discharge the patient was able to walk without support. This favorable outcome was in line with the patient’s low mEGOS value at the onset of neurological symptoms.

In our opinion, this case gives the opportunity to discuss several crucial points regarding the possible neurological complications of SARS-CoV-2 infections. From a clinical point of view, there were some confounding factors: indeed, autonomic impairment could have been attributed to other coexisting factors, such as diabetic neuropathy, patient’s age, and muscular weakness due to SARS-CoV-2 infection. SARS-CoV-2 infection itself has also been temporally related to dysautonomic symptoms (Shouman et al. 2021). However, results of neurophysiological study, CSF findings, and response to intravenous immunoglobulin treatment support the diagnosis of GBS, which is included among the possible neurological manifestations after SARS-CoV-2 infection (Ahmad and Rathore, 2020; Sriwastava et al. 2021).

In our case, electrophysiological pattern confirmed the diagnosis of GBS: in fact, while in diabetic neuropathy, the damage is typically axonal and sensory (Feldman et al. 2019); in our patient, the electrophysiological findings, according to Hadden’s criteria (Hadden et al. 1998), allowed to identify an acute inflammatory demyelinating neuropathy (AIDP), probably superimposed over a diabetic neuropathy (Table 1). Hyperproteinorrachia was not severe, probably due to the spinal tap performed quite close to symptoms onset (Leonhard et al. 2019).

Interestingly, similar short interval between infection and GBS onset has been previously reported in other patients with SARS-CoV-2-related GBS (Toscano et al. 2020). In these cases, classifiable more as parainfectious than postinfectious GBS, the pathogenetic mechanism could be ascribed to an aberrant immune response rather than the typical molecular mimicry-based mechanism.

Limits of our report are that we have not carried out specific electrophysiological tests for dysautonomia; moreover, in our case, serology for SARS-CoV-2 IgM was negative. The presence of IgG alone could be due to seroconversion already occurred from IgM to IgG at the time of the sample collection. Anyway, so far, three different types of seroconversion have been described (Long et al. 2020), one of them consisting in seroconversion to IgG preceding the IgM one.

In this case, dysautonomic symptoms represented the main feature of GBS. Although it does not fulfill all GBS clinical criteria (Fokke et al. 2014), a variant of GBS presenting as acute autonomic neuropathy has already been published (Koike et al. 2013). Moreover, recently an autonomic dysfunction preceding acute motor axonal neuropathy (AMAN) associated with COVID-19 has been reported (Ghosh et al. 2020); as opposed to this case, in our patient, autonomic dysfunction was the only clinical presentation of peripheral neuropathy, whose autoimmune mechanism was confirmed by CSF results and response to immunomodulating therapy.

In conclusion, we believe that our case of pure dysautonomic presentation of GBS in a patient with SARS-CoV-2 infection is important because it might alert clinicians to search for GBS after SARS-CoV-2 infection, even in cases of isolated dysautonomic symptoms, especially when there are confounding factors such as old age and diabetes.
